# A multi-Kalman filter-based approach for decoding arm kinematics from EMG recordings

**DOI:** 10.1186/s12938-022-01030-6

**Published:** 2022-09-03

**Authors:** Hend ElMohandes, Seif Eldawlatly, Josep Marcel Cardona Audí, Roman Ruff, Klaus-Peter Hoffmann

**Affiliations:** 1 Center of Informatics Science, Nile University, Giza, Egypt; 2 Mathematical and Computer Science, Heriot-Watt, Dubai, United Arab Emirates; 3 Computer and Systems Engineering Dept, Faculty of Engineering, Ain Shams University, Cairo, Egypt; 4 Faculty of Media Engineering and Technology, German University in Cairo, Cairo, Egypt; 5 Department of Medical Engineering and Neuroprostheses, Fraunhofer IBMT, Sulzbach, Germany

**Keywords:** Kalman filter, Decoding, EMG, Prosthetic arms

## Abstract

**Background:**

Remarkable work has been recently introduced to enhance the usage of Electromyography (EMG) signals in operating prosthetic arms. Despite the rapid advancements in this field, providing a reliable, naturalistic myoelectric prosthesis remains a significant challenge. Other challenges include the limited number of allowed movements, lack of simultaneous, continuous control and the high computational power that could be needed for accurate decoding. In this study, we propose an EMG-based multi-Kalman filter approach to decode arm kinematics; specifically, the elbow angle (**θ**), wrist joint horizontal (**X**) and vertical (**Y**) positions in a continuous and simultaneous manner.

**Results:**

Ten subjects were examined from which we recorded arm kinematics and EMG signals of the biceps, triceps, lateral and anterior deltoid muscles corresponding to a randomized set of movements. The performance of the proposed decoder is assessed using the correlation coefficient (CC) and the normalized root-mean-square error (NRMSE) computed between the actual and the decoded kinematic. Results demonstrate that when training and testing the decoder using same-subject data, an average CC of 0.68 ± 0.1, 0.67 ± 0.12 and 0.64 ± 0.11, and average NRMSE of 0.21 ± 0.06, 0.18 ± 0.03 and 0.24 ± 0.07 were achieved for **θ**, **X**, and **Y**, respectively. When training the decoder using the data of one subject and decoding the data of other subjects, an average CC of 0.61 ± 0.19, 0.61 ± 0.16 and 0.48 ± 0.17, and an average NRMSE of 0.23 ± 0.07, 0.2 ± 0.05 and 0.38 ± 0.15 were achieved for **θ**, **X**, and **Y**, respectively.

**Conclusions:**

These results suggest the efficacy of the proposed approach and indicates the possibility of obtaining a subject-independent decoder.

**Supplementary Information:**

The online version contains supplementary material available at 10.1186/s12938-022-01030-6.

## Background

With the increase in number of amputees worldwide, the need for the development of a better and more functional replacement arises [1]. Many attempts and developments were made throughout history starting with passive and cosmetic prostheses with no functionality, moving to body-powered prosthetics [2, 3], and reaching myoelectric prostheses that were introduced in early 1950’s [4]. Myoelectric prostheses represent a gate to developing a functional prosthesis with a step closer to the natural limb. Such devices use signals recorded from the nervous system, such as electromyography (EMG) signals to control the prosthesis. Using EMG signals has shown success in this application as they can be recorded non-invasively with no risks imposed. EMG signals have also been shown to carry neural information on the motor intention similar to direct nerve recordings, which indicates that movements could be decoded from EMG signals [5, 6].

Although this type of prosthesis has been first introduced long time ago, there have been significant efforts to date to develop advanced control techniques that could provide more accurate control of the prosthesis [7]. Many techniques employed pattern recognition methods to control the prosthesis in which signals are being classified to one of limited classes of movements in a discrete form [7–9]. The limited functionality and low reliability of these methods motivate the use of statistical techniques that could provide more promising solutions as they overcome some of the pattern recognition limitations. For instance, multiple approaches investigating simultaneous, proportional and continuous control using regression-based techniques have been introduced [10, 11]. Unlike pattern recognition techniques, such techniques do not map discrete movements, but rather calculate an estimation for each movement degree-of-freedom (DOF). This allows simultaneous and independent control for the user.

Other techniques were also used in which proportional control is employed which uses EMG signals to control speed or force of movement according to the recorded EMG, threshold control and onset analysis [12]. This approach can detect the onset and offset of muscle contraction. Although such approach demonstrated accurate performance, it has a high computational complexity which negatively impacts the feasibility of using it in real-time control.

Representing a plausible alternative control technique, the Kalman filter is considered one of the most powerful statistical methods. It has been widely used in various applications, such as tracking moving objects [13, 14], building a fuzzy inference system together with generalized neural networks [15], as well as using it in image and motion analysis to estimate pixels depth and depth uncertainty [16]. In addition, the Kalman filter has been widely used in brain–computer interface (BCI) applications. It has been demonstrated to control the motion of a mouse cursor using neural signals recorded from motor cortex [17]. It was also used in inferring hand motion from multi-cell recordings from the motor cortex [18]. The Kalman filter showed promising results in each application with high capability of state estimation, and low computational power. However, previous studies have demonstrated low prediction accuracy when using the Kalman filter in decoding EMG signals [11]. Using manually tuned Kalman filter or the more computationally expensive non-linear versions of the Kalman filter have been demonstrated to enhance the decoding accuracy [19, 20]. In some of these studies, a set of restricted movements were only allowed for the subjects to perform, which might impact the ability of the filter to model naturalistic movements. Finally, these studies focused on training subject-dependent Kalman filters that are specifically trained for each subject.

In this paper, we propose a multi-Kalman filter-based decoding scheme to estimate arm elbow angle **θ**, and **X** and **Y** positions of the wrist from surface EMG signals captured from 4 muscles group biceps, triceps lateral and anterior deltoid. We introduce a novel setup that we used to record the random and complex movements kinematics and EMG signals simultaneously from 10 different subjects. The subjects were instructed to perform sets of movements in 2D plane in a random form without restricting the movements to a specific sequence. In our approach, we first train multiple Kalman filters using pairs of parameters (i.e., (**θ**, **X**)**,** (**θ**, **Y**) and (**X, Y)**) to obtain their parameters and then use them to decode the EMG signal. The best filter for each parameter is identified and subsequently used in decoding test data for each subject. We examined the performance of the approach in both a subject-dependent and subject-independent manner, where in the latter, Kalman filters were trained using EMG data of some subjects and tested on completely different subjects. Our results demonstrate the capability of the introduced approach to decode a wide range of angles and coordinates position in a more continuous form in both the subject-dependent and subject-independent cases. It showed that it could withstand the randomness and the sudden changes in the data set, resulting in an enhanced performance with minimal processing. This could allow prosthesis control in a smoother, continuous and simultaneous manner.

## Results

### Recorded data

**Fig. 1:**
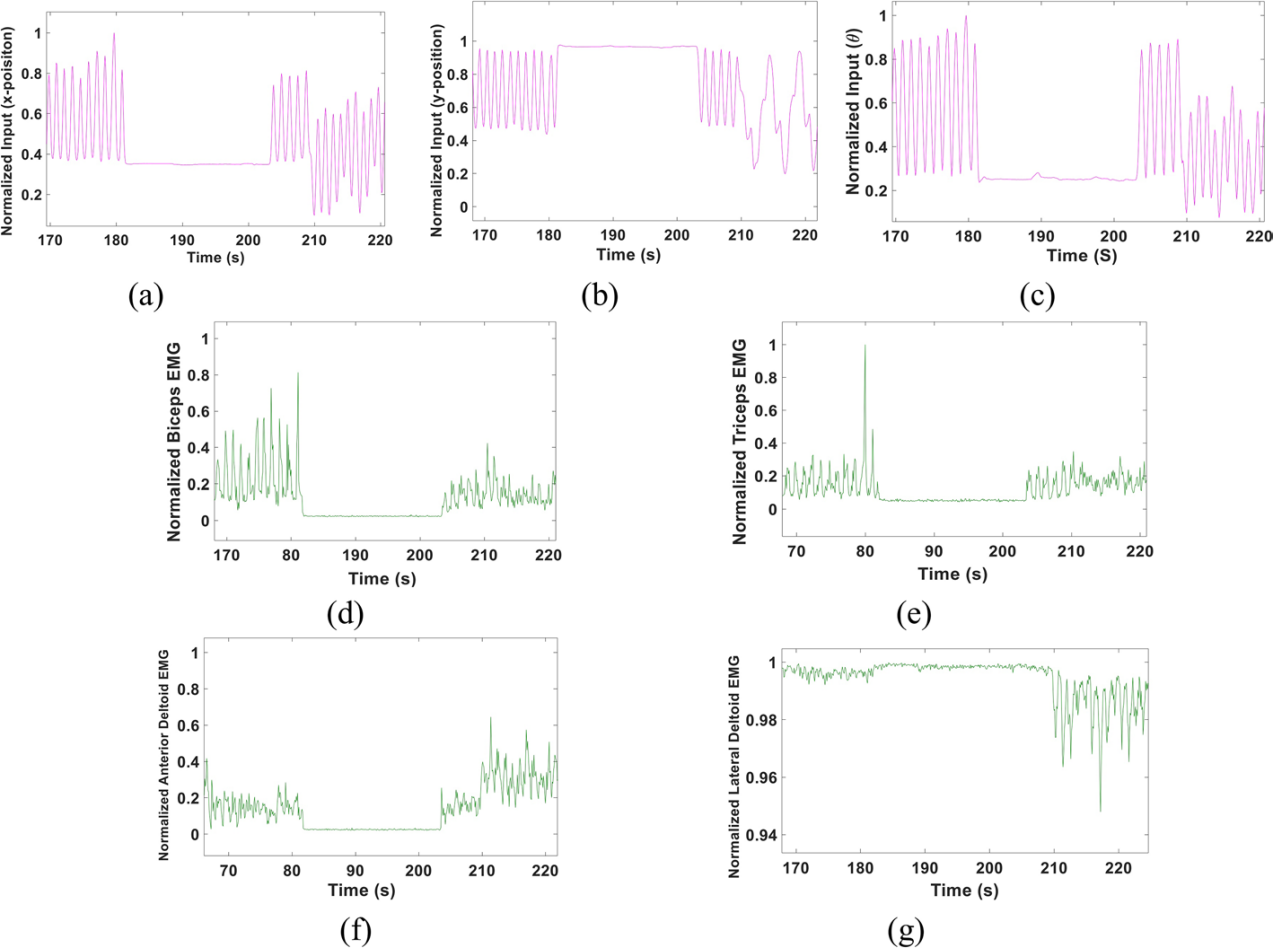
Sample data set of elbow angles and corresponding EMG recorded from biceps, triceps, Anterior and lateral Deltoid muscles. **a** X-Position of the wrist. **b** Y-position of the wrist. **c** Elbow angle θ. **d** Corresponding Normalized Biceps EMG. **e** Corresponding Normalized Triceps EMG. **f** Corresponding Normalized Anterior Deltoid EMG. **g** Corresponding Normalized Lateral Deltoid EMG zoomed in to show the change in signal

We examined the performance of the proposed multi-Kalman filter approach using 20 sets of data. Two sessions were recorded from each subject, where the first session data set was used to train the Kalman filters and obtain the four parameters **A**, **H**, **Q** and **W** (see Additional file 1: Fig. S1), and the other session data set was used for testing. Figure 1 shows samples of **X–Y** position, elbow angle **θ** as well as the corresponding EMG signals. The figures demonstrate how a change in arm movement results in a clear muscle activity. For instance, the figures show a clear synchronization between the biceps EMG (Fig. 1d) and **X** (Fig. 1a) and **θ** (Fig. 1c) positions parameters.

### Subject-dependent performance

**Fig. 2:**
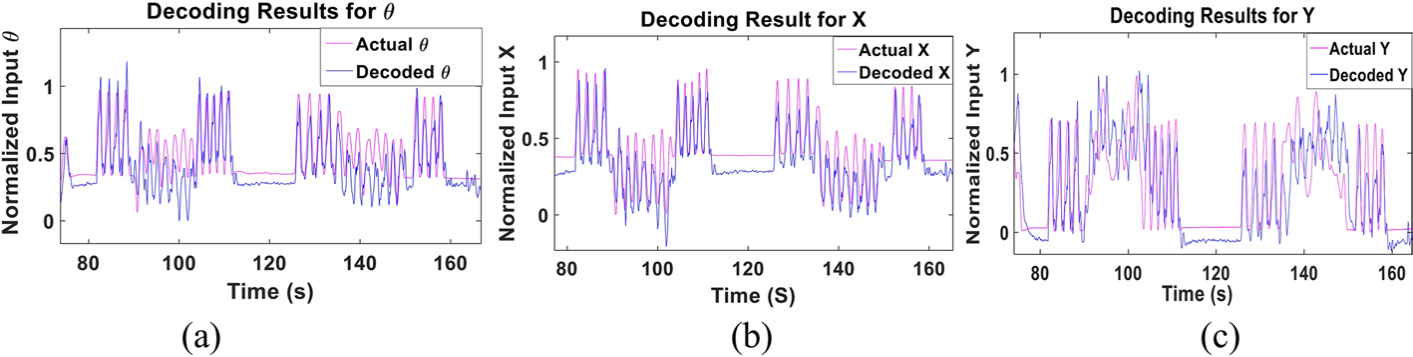
Sample of the multi-Kalman filter performance showing (**a**) decoding result for **θ**, (**b**) **X** and (**c**) **Y**

**Fig. 3:**
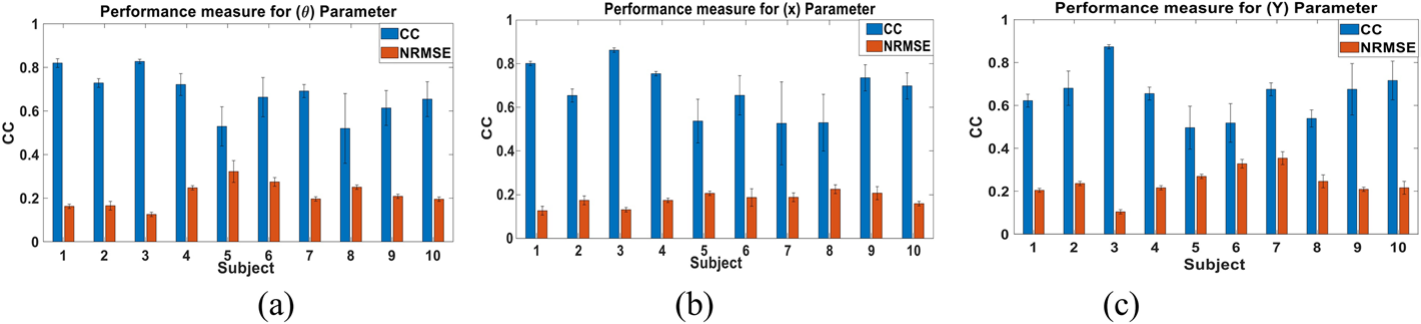
CC and NRMSE for each subject for each of (**a**) **θ**, (**b**) **X** and (**c**) **Y** the subject-dependent test

We first examined the performance of the proposed parameters combination Kalman filter approach when trained and tested using the data of each subject. The data set of each subject was randomly split to 75% training data and 25% testing data for 10 different folds. Figure 2 shows a sample of the decoded and actual state for **θ**, **X** and **Y** for the subject with the best performance (Subject 3). The results show that the proposed approach is able to follow the actual input and its transitions accurately, even when a sudden change in the movement occurs. The figure also demonstrates that the pattern of change for **θ** and **X** is similar, representing the nature of the movements done by the subject. Figure 3 shows the CC and NRMSE obtained for each subject for the **θ**, **X** and **Y** parameters. The parameters combination approach decoded the test data set with a mean CC, computed across subjects, of 0.68 ± 0.1, 0.67 ± 0.12 and 0.64 ± 0.11, and with mean NRMSE of 0.21 ± 0.06, 0.18 ± 0.03 and 0.24 ± 0.07 for **θ**, **X** and **Y**, respectively. Results indicate that the Kalman filter was able to decode **θ** for most subjects with high performance and had a lower performance for 20% of subjects. Tables 1 and 2 show the detailed CC and NRMSE values for each subject.

**Table 1:** Detailed CC for each subject for θ, X and Y for the subject-dependent test

	Subject number										
	1	2	3	4	5	6	7	8	9	10	Mean
θ	0.82 ± 0.02	0.73 ± 0.02	0.83 ± 0.01	0.72 ± 0.05	0.53 ± 0.09	0.66 ± 0.09	0.69 ± 0.03	0.52 ± 0.16	0.61 ± 0.08	0.65 ± 0.08	0.68 ± 0.1
X	0.8 ± 0.01	0.65 ± 0.03	0.86 ± 0.01	0.75 ± 0.01	0.54 ± 0.10	0.65 ± 0.09	0.53 ± 0.19	0.52 ± 0.13	0.73 ± 0.06	0.7 ± 0.06	0.67 ± 0.12
Y	0.62 ± 0.03	0.68 ± 0.08	0.87 ± 0.01	0.65 ± 0.03	0.5 ± 0.10	0.52 ± 0.09	0.67 ± 0.03	0.54 ± 0.04	0.67 ± 0.12	0.72 ± 0.09	0.64 ± 0.11

**Table 2:** Detailed NRMSE for each subject for θ, X and Y for the subject-dependent test

	Subject number										
	1	2	3	4	5	6	7	8	9	10	Mean
θ	0.16 ± 0.01	0.16 ± 0.02	0.12 ± 0.01	0.25 ± 0.01	0.32 ± 0.05	0.27 ± 0.02	0.2 ± 0.01	0.25 ± 0.01	0.21 ± 0.01	0.19 ± 0.01	0.21 ± 0.06
X	0.12 ± 0.02	0.17 ± 0.02	0.13 ± 0.01	0.17 ± 0.01	0.21 ± 0.01	0.19 ± 0.04	0.19 ± 0.02	0.22 ± 0.02	0.21 ± 0.03	0.16 ± 0.01	0.18 ± 0.03
Y	0.20 ± 0.01	0.24 ± 0.01	0.1 ± 0.01	0.22 ± 0.01	0.27 ± 0.01	0.33 ± 0.02	0.35 ± 0.03	0.25 ± 0.03	0.21 ± 0.01	0.22 ± 0.03	0.24 ± 0.07

### Subject-dependent performance using biceps and triceps signals only

**Fig. 4:**
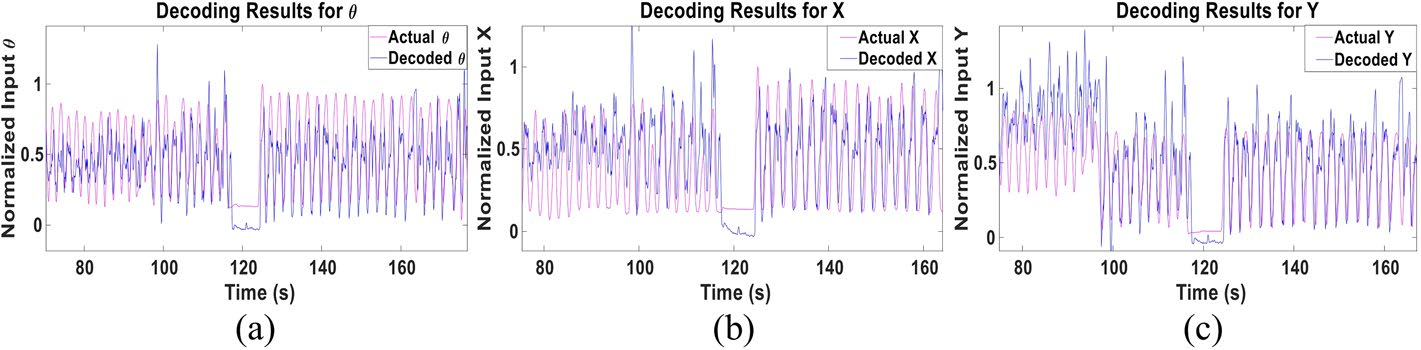
Sample of the multi-Kalman filter performance when using signals from the biceps brachii and triceps brachii only showing (**a**) decoding result for **θ**, (**b**) **X** and (**c**) **Y**

**Fig. 5:**
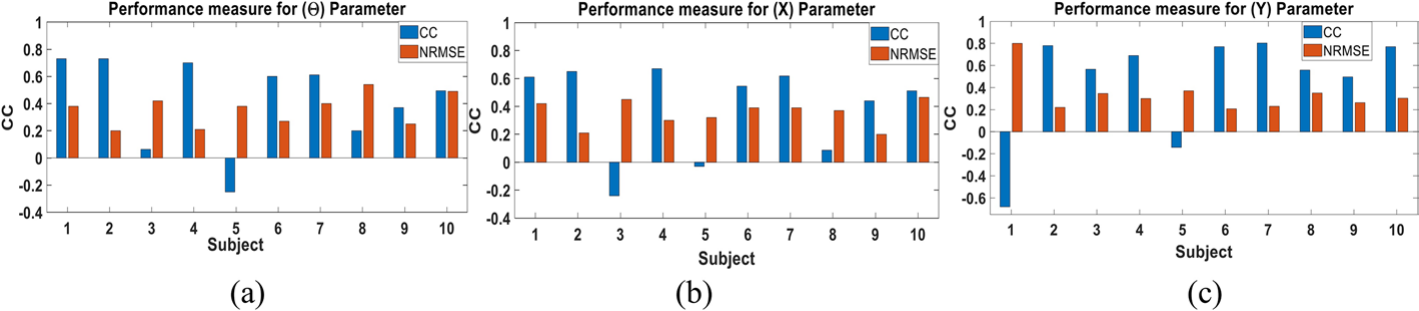
CC and NRMSE for each subject when using signals from the biceps brachii and triceps brachii only for each of (**a**) **θ**, (**b**) **X** and (c) **Y** the subject-dependent test using biceps and triceps EMG signals only

To assess whether all four sets of electrodes are needed or using signals recorded from the biceps and triceps muscles would be sufficient, we examined the performance of the Kalman filter when using EMG signal from biceps brachii and triceps brachii only. The data set of each subject was randomly split to 75% training data and 25% testing data. Figure 4 shows a sample of the decoded and actual state for **θ**, **X** and **Y** for the subject with the best performance using this set of electrodes (Subject 2). While the decoded parameters appear to be able to track their corresponding actual values, this occurs at a lower accuracy compared to using all four sets of electrodes. Figure 5 shows the CC and NRMSE obtained for each subject for the **θ**, **X** and **Y** parameters. The parameters combination approach decoded the test data set with a mean CC, computed across subjects, of 0.42 ± 0.32, 0.386 ± 0.32 and 0.46 ± 0.488, and with mean NRMSE of 0.35 ± 0.12, 0.351 ± 0.09 and 0.338 ± 0.17 for **θ**, **X** and **Y**, respectively. Results indicate that the Kalman filter performance dropped significantly for 80% of subjects, while it remained unaffected for the other 20% compared to using all four sets of electrodes. These results support our observation, since for movements, such as shoulder flexion and extension, the deltoid muscles are activated.

### Subject-independent leave-one-out performance

**Fig. 6:**
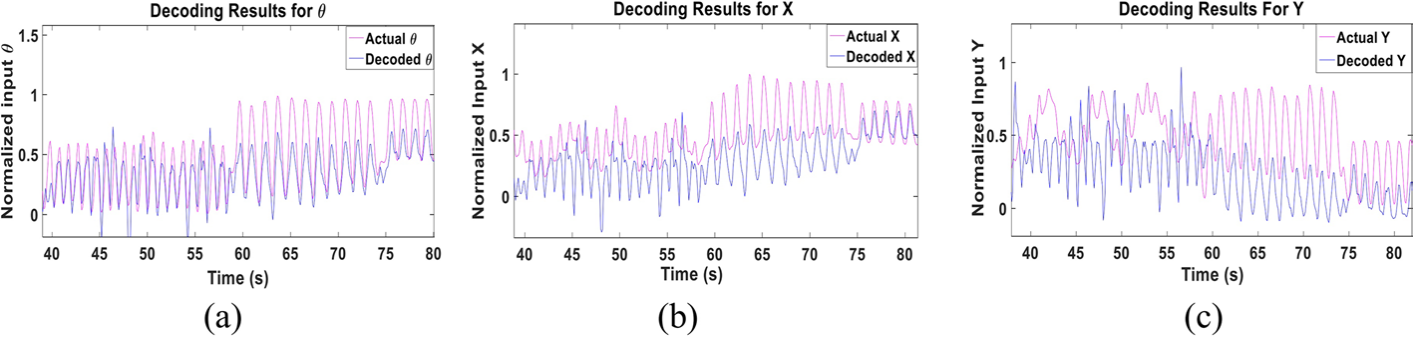
Sample of multi-Kalman filter performance showing (**a**) decoding result for **θ**, (**b**) **X** and (**c**) **Y** for the subject-independent leave-one-out test

**Fig. 7:**
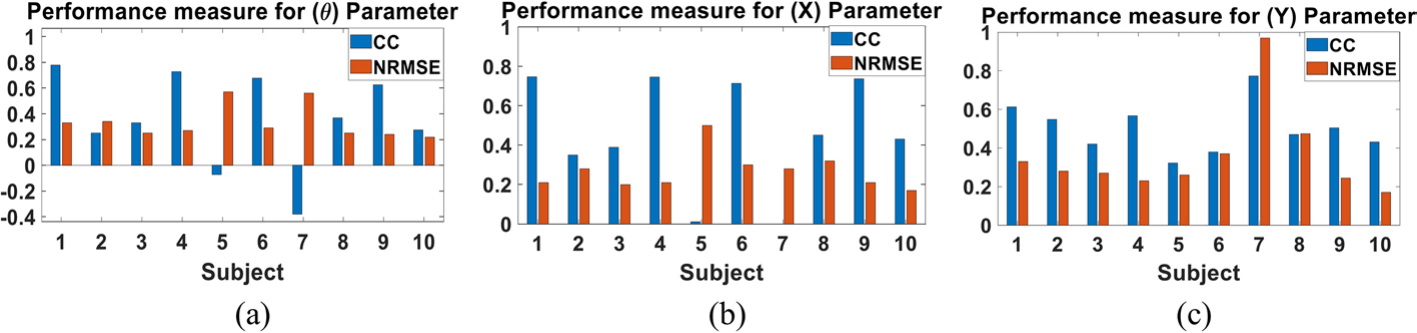
CC and NRMSE for each subject for each of (**a**) **θ**, (**b**) **X** and (**c**) **Y** for the subject-independent leave-one-out test

We next examined the performance of the multi-Kalman filter approach when trained using the data of 9 subjects and tested using the data of the left-out 10^th^ subject. This test was performed to examine the performance of Kalman filter if training was done using more diverse data sets that could completely eliminate the need for recording training data from each subject. Figure 6 shows a sample of the decoded and actual states for **θ**, **X** and **Y** of Subject 1. The figure demonstrates similarity, albeit lower than that achieved in the subject-dependent test, that is quantified in Fig. 7 for all subjects showing the CC and NRMSE for the three parameters. A mean CC of 0.61 ± 0.19, 0.61 ± 0.16 and 0.48 ± 0.17 and mean NRMSE of 0.27 ± 0.07, 0.2 ± 0.05 and 0.38 ± 0.15 were achieved for **θ**, **X** and **Y**, respectively. Despite such drop, a slight improvement in the performance for some subjects can be detected as well, as in Subject 5, for example. Tables 3 and 4 show the detailed CC and NRMSE for each subject.

**Table 3:** Detailed CC for each subject for θ, X and Y for the subject-independent leave-one-out test

	Subject Number										
	1	2	3	4	5	6	7	8	9	10	Mean
θ	0.78	0.25	0.33	0.73	-0.07	0.677	-0.38	0.422	0.663	0.581	0.61 ± 0.19
X	0.75	0.35	0.39	0.75	0.01	0.714	0.194	0.575	0.674	0.524	0.61 ± 0.16
Y	0.61	0.55	0.42	0.57	0.32	0.379	0.653	0.574	0.387	0.563	0.48 ± 0.17

**Table 4:** Detailed NRMSE for each subject for **θ**, **X** and **Y** for the subject-independent leave-one-out test

	Subject Number										
	1	2	3	4	5	6	7	8	9	10	Mean
θ	0.33	0.34	0.25	0.27	0.57	0.29	0.2	0.32	0.22	0.19	0.23 ± 0.07
X	0.21	0.28	0.20	0.21	0.50	0.30	0.16	0.24	0.21	0.18	0.2 ± 0.05
Y	0.33	0.28	0.27	0.23	0.26	0.37	0.28	0.47	0.26	0.45	0.38 ± 0.15

### Subject-independent performance based on best subject training

**Fig. 8:**
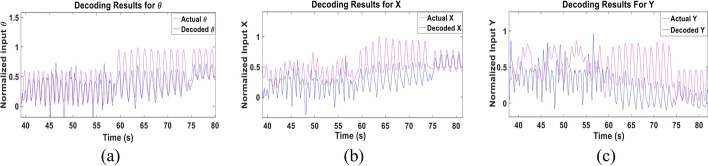
Sample of the multi-Kalman filter performance showing (**a**) **θ**, (**b**) **X** and (**c**) **Y** for the subject-independent testing with the Kalman filters using data of Subject 3

**Fig. 9:**
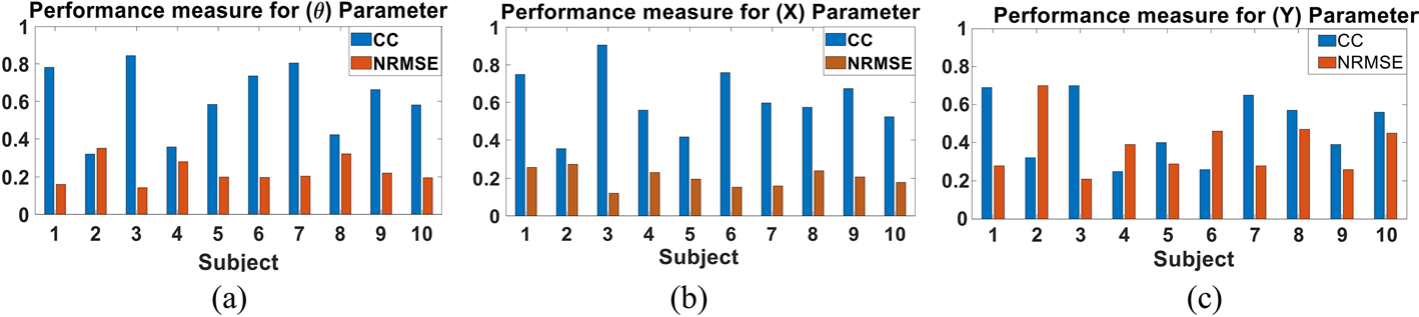
CC and NRMSE for each subject for each of (**a**) **θ**, (**b**) **X** and (**c**) **Y** for the subject-independent testing with the Kalman filters using data of Subject 3

In this test, we examined the performance of the approach when we train using data from the subject with best subject-dependent performance and test using data of each of the other subjects. The data set of Subject 3 was used for training in this case given the elevated performance obtained using the proposed approach for this subject compared to other subjects as demonstrated in Fig. 3. Figure 8 illustrates a sample of the decoded and actual state for **θ, X** and **Y** of Subject 1. Figure 9 shows the CC and NRMSE obtained for each subject for each of the 3 parameters. The overall performance showed a drop compared to the subject-dependent test, with mean CC of 0.61 ± 0.19, 0.61 ± 0.16 and 0.48 ± 0.17 and mean NRMSE 0.23 ± 0.07, 0.2 ± 0.05 and 0.38 ± 0.15 for **θ, X** and **Y**, respectively. Despite such drop, a slight improvement in the performance for some subjects can be detected as well, as in Subject 5, for example. Tables 5 and 6 show the detailed CC and NRMSE for each subject.

**Table 5:** Detailed CC for each subject for **θ**, **X** and **Y** for the subject-independent testing with the Kalman filters using data of Subject 3

	Subject Number										
	1	2	3	4	5	6	7	8	9	10	Mean
θ	0.78	0.32	0.84	0.36	0.58	0.74	0.8	0.42	0.66	0.58	0.61 ± 0.19
X	0.75	0.35	0.9	0.56	0.43	0.77	0.6	0.57	0.67	0.52	0.61 ± 0.16
Y	0.69	0.32	0.7	0.25	0.4	0.26	0.65	0.57	0.39	0.56	0.48 ± 0.17

**Table 6:** Detailed NRMSE for each subject for **θ**, **X** and **Y** for the subject-independent testing with the Kalman filters using data of Subject 3

	Subject number										
	1	2	3	4	5	6	7	8	9	10	Mean
θ	0.16	0.35	0.14	0.28	0.2	0.2	0.2	0.32	0.22	0.19	0.23 ± 0.07
X	0.25	0.27	0.12	0.23	0.19	0.15	0.16	0.24	0.21	0.18	0.2 ± 0.05
Y	0.28	0.70	0.21	0.39	0.29	0.46	0.28	0.47	0.26	0.45	0.38 ± 0.15

### Comparison to other Kalman filter approaches

**Fig. 10:**
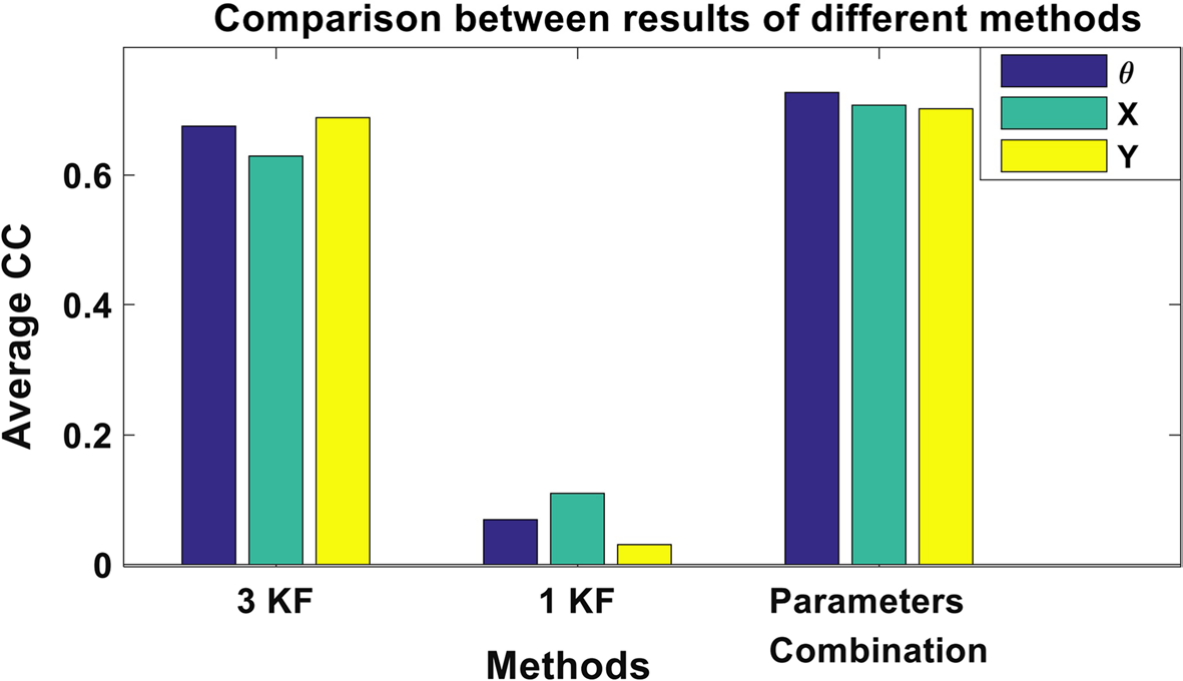
Average CC for three parameters when using 3 Kalman filters, 1 Kalman filter and Parameters Combination approaches

We examined the performance of the multi-Kalman filter proposed approach in comparison to the other two approaches (the single Kalman filter approach and the three Kalman filter approach). Figure 10 shows the result of different attempts to construct a Kalman filter with one, two and three parameters, with the average CC for each parameter in each method for the subject-dependent testing. Results demonstrate that the proposed parameters combination approach achieves the best performance. Parameters combination resulted in a mean CC of 0.698, 0.675 and 0.652 compared to 0.069, 0.109 and 0.0308 for the single Kalman filter approach and 0.67, 0.629 and 0.688 for the three Kalman filter approach, for **θ**, **X** and **Y** position, respectively.

## Discussion

Enhancing movement kinematics decoding from EMG signals represents one crucial step toward the development of prosthetics with naturalistic movements. Here, we aimed to develop a multi-Kalman filters approach that results in the best performance for each decoded movement parameter. The proposed approach showed enhanced performance when tested in subject-dependent and subject-independent manners. The proposed technique is capable of overcoming measurement noise and shows the possibility of across-subjects decoding.

There have been multiple efforts employing a variety of techniques for movement decoding from EMG signals. For instance, Loconsole et al. used time-delayed neural network (TDNN) for online torque prediction and control of robot joints, using sEMG signals as the input to the model. The achieved results demonstrate the possibility of using this approach to support patients with movements difficulty [21]. However, in such setup, it is unclear if this model can predict direct kinematics, such as elbow/finger angles, or a given position of arm. In addition, the trajectory of the movement was limited and might only be suitable for rehabilitation rather than prosthesis control. Natsakis et al. attempted to estimate the kinematics from the elbow down using EMG signals measured from biceps, triceps, deltoid and brachioradialis muscles group [22]. In this study, a neural network model was used in the estimation process. However, the study does not show if this model can be used for amputees with missing brachioradialis muscle. In addition, the approach was only examined using limited repetitive set of movements without reporting the performance if the subjects were instructed to move in no planned pattern. Finally, Kapelner et al. attempted to predict wrist kinematics using both EMG signals and neural features extracted from the decomposition of recorded EMG. A simple linear regression model is used in this approach for prediction. However, one limitation of this study is including only single DOF contractions [23].

Given the limitations of the aforementioned studies, the Kalman filter has been utilized in our method given its known strength in prediction problems for continuous systems [24–26]. Multiple studies have introduced the usage of Kalman filter for movement decoding, however, using motor cortex neuronal activity recorded invasively [17, 27, 28]. Such technique could provide access to the activity of single neurons which results in accurate decoding of movement intention. However, it is still far from being applied in practice given the complications that might be associated with electrode implantation surgeries. Therefore, using EMG could provide better alternative for which different decoding schemes have been proposed for a similar purpose.

In our study, we have proposed solution that could address some of the limitations in prosthesis control. The Kalman filter is ideal for dynamic systems for which it is able to iteratively decode the signals using consecutive data inputs [29, 30]. This allows it to quickly estimate the true value while taking into consideration the uncertainty of the given data. Another advantage of Kalman filters is that they do not require significant memory resources as they only need the previous state for the next estimation [31]. This makes Kalman filters suitable for real-time applications, such as prosthesis control. It could be applied to time-domain EMG without the need to extract other frequency-domain features (see Additional file 1: Figs. S2–S5). It would not require high computational power compared to using neural networks and deep learning techniques. Moreover, it can overcome the measurement (EMG signal) noise, which is critical in such application given that EMG signals are noisy [32], especially when recorded non-invasively. We have also shown the possibility of training using one subject’s data and using the model to decode the movement for a completely different subject.

This work could be extended to take into account the dynamics of EMG signals when the muscle is in fatigue, as we were attentive to give intervals of rest to our subjects to avoid changes in the muscle activity. Thus, the behavior of the proposed approach cannot be predicted if, for a given movement, a different EMG signal is introduced for decoding. This might not be practical for clinical setups. This could be alleviated using an adaptive Kalman filter-based decoder, such as the adaptive Kalman filter [33]. Another issue is that the Kalman filter employed in our approach assumes a linear relationship between the movement and the recorded EMG signal. While it was successful in decoding the movement with acceptable performance, it is expected that using a non-linear filter such as the unscented Kalman filter would be able to decode with better performance, however, with a relatively higher computational complexity [34]. Finally, in this paper, we recorded both movement kinematics and EMG signals using a rather limited setup. With the many new advanced hardware for EMG recording and movement tracking being introduced, it makes us expect that the usage of such technologies might enhance the noise filtration and provide more accurate readings. In addition, it would result in less delay in the simultaneous recording of both EMG and kinematics. It could also allow online decoding of the EMG signal and investigating its behavior. With the suggestions above, the approach and results demonstrated in this paper could provide means for prosthesis control improvement.

## Conclusions

In this paper, we introduced an EMG decoding scheme for arm kinematics in 2D plane. Our approach consists of 4 phases: data recording, preprocessing of data, identifying the best combination of parameters for the decoding process and training the Kalman filter using the training data set. The proposed algorithm was tested on multiple subjects, where one recording session is used for training and the other recorded session is used for testing the performance of the algorithm. We also examined the performance when the training data of one subject is used to train the algorithm and subsequently use the trained decoder to decode the data of other subjects. Results showed the ability of the proposed multi-Kalman filter approach to decode kinematics in 2D plane using EMG signals recorded from 4 muscle group. We also demonstrated the success in training the filter using data from one subject and decoding the data of another subject. Our results demonstrate the ability of the proposed approach to decode arm kinematics in a continuous and simultaneous form which could result in a smoother movement prosthesis than the ones available nowadays. Such an algorithm would enable the introduction of a new family of prostheses that can behave in a closer form to a natural arm.

## Methods

### Experimental setup

Ten healthy, right-handed subjects participated in this study (five males, five females, age range 21–33). None of them had any history of neuromuscular disorder and all of them provided written consent to the procedures. The objective of the experiment was to record EMG signals simultaneously with the corresponding kinematics of the arm. Subjects had 9 electrodes connected to 4 muscle groups: biceps, lateral triceps, anterior deltoid and lateral deltoid for EMG recording. The 9^th^ electrode was used as reference and was positioned at the center of the scapula. Prior to attaching the electrodes, subjects’ skin was wiped using an isopropyl alcohol pad for removal of oils and surface residues to increase recording electrodes impedance [35]. The skin was allowed to air dry for a few seconds before placing the gel electrodes. Electrodes placement was done following recommendations in [36].

**Fig. 11:**
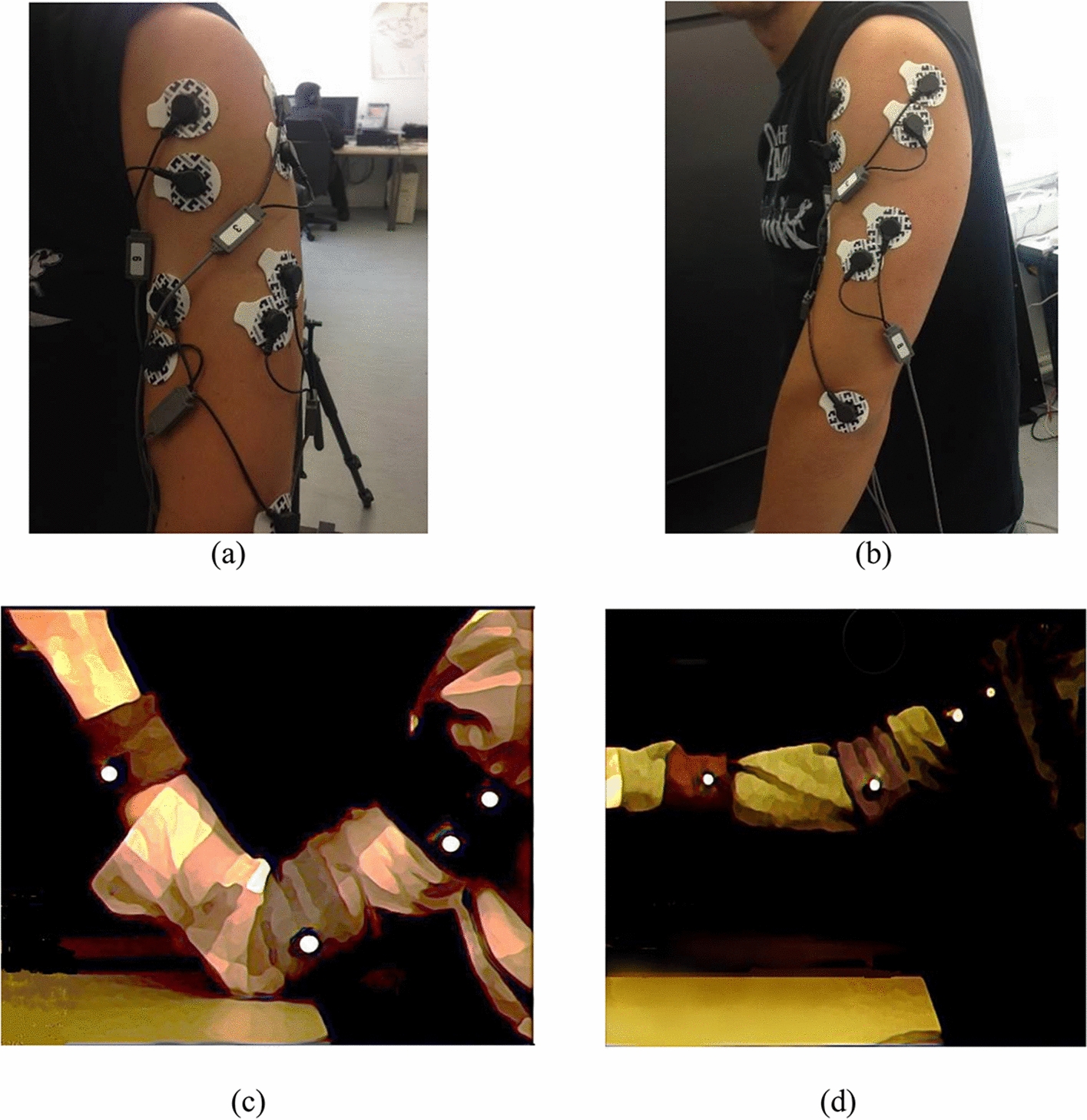
**a** Subject’s arm showing electrodes connected to biceps and deltoid muscle groups. **b** Subject’s arm showing electrodes connected to triceps deltoid and reference. **c** Subject’s arm with markers attached to the arm position matching movement number 1. **d** Sample of the subject’s arm movement number 2

Four markers were used: two near the surgical neck, one at the elbow joint and one at the carpal (wrist) joint, for movement tracking, as illustrated in Fig. 11. The markers were fixed on wearable bands. All subjects used the same band without changing the location of the markers shown in Fig. 11c, .

Participants were instructed to make sets of movements in 2D space with no specific order or frequency. The movements were classified as follows:

1. Flexion and extension of forearm. This movement takes place while resting the elbow joint on a fixed table or without resting it on the table. This leads to a change of the elbow angle while using mainly the biceps brachii and triceps brachii.
2. Shoulder flexion/extension with or without forearm flexion/extension. This movement reflect an imitation of reaching out to an object.
3. Resting the arm on the table to avoid muscle fatigue. Subjects were instructed to rest anytime they feel tired until well-rested. The average time per rest was estimated at 2–3 s.

**Fig. 12:**
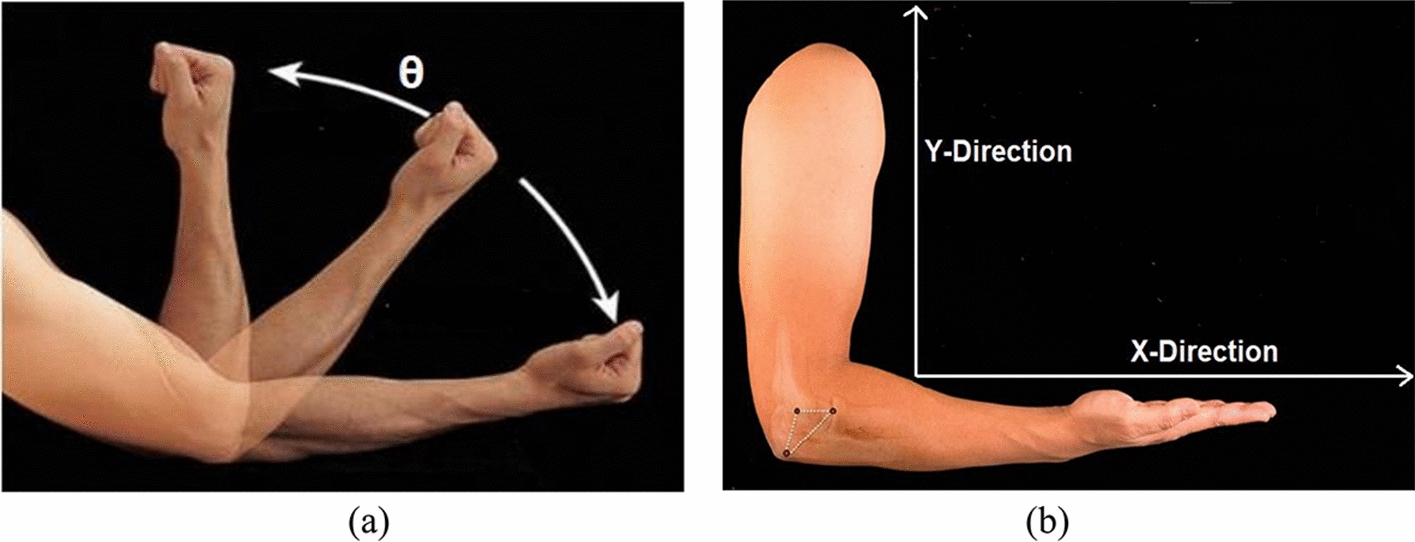
Visual illustration of the plane of movements. **a** Elbow angle that was captured. **b** *x*–*y* plane of arm movement

Figure 12 shows a detailed description of the movements described. Subjects were not instructed to avoid supination and/or pronation of forearm during movements. All subjects participated in two sessions, separated with 5 to 10 min breaks. The sessions were 400 and 200 s long, respectively.

### Data recording

SIMI reality motion system (SIMI, Germany) was used as an environment for motion capturing using an image analysis technique. Video recording was made using a Basler A60xf EMVA (Basler Technologies, Ahrensburg, Germany) with frame rate of 15 frames per second and saved using synchronized industrial cameras. The hardware used for EMG capturing was Noraxon TelyMyo 2400 T (Noraxon, USA, Inc.). A national instrument card NI USB-6225 (National Instruments Corporation, Austin, USA) was also used for the digitization process of the signals. EMG signals were recorded simultaneously with the video of motion through both SIMI and Noraxon platforms.

At the end of each session, the tracking system was used to analyze motion using marker-based automatic tracking after proper calibration. The resulting data file for each subject contained the time samples, the elbow angle, the four EMG signals, and the **X** and **Y** positions of wrist marker. Using the positions of each of the remaining 3 markers, we calculated the elbow angle ${\varvec{\uptheta}}$ using an embedded function in SIMI software.

### Data pre-processing

The EMG signal recordings underwent filtration process on two stages, the first stage was using a built-in hardware 1st order high-pass filter with 10 Hz ± 10% cutoff. An additional 8th order Butterworth/Bessels low-pass anti-alias filter set to 500 Hz ± 2% cutoff was applied [37]. The second stage was smoothing the signal using root mean square (RMS), which acts as a linear envelope for the EMG signal. The RMS squares the raw signal which is considered a measurement of the power of the signal [38, 39]. All the collected signals were then normalized by subtracting the minimum value and then dividing each sample by the maximum value of all samples:

1 $$\mathbf{h}\left(t\right)=\frac{\left(\mathbf{k}-\mathrm{min}(\mathbf{k})\right)}{\mathrm{max}\left(\mathbf{k}-\mathrm{min}(\mathbf{k})\right)}$$

where **k** denotes the *X-axis* position, *Y-axis* position or the elbow joint angle $\theta$. In addition, all EMG signals were multiplied by a constant factor of 10^3^ and measured kinematics was multiplied by factor of 10^2^ to increase their amplitude as specified for the Noraxon device.

### Data analysis

Our objective is to estimate the kinematic state of the arm at a specific instance of time *n*. The state is defined by ${\widehat{\mathbf{r}}}_{n}=[{\varvec{\uptheta}},\mathbf{X},\mathbf{Y}]$, representing the elbow joint angle, *X*-position and *Y*-position of the wrist joint, respectively, at given time *T* = $\Delta$ *t* × *n*, where $\Delta$ *t* is our time window which was set to 100 ms. The three parameters are used to describe the movement described in Sect. 5.1. These parameters are independent as the same ($\mathbf{X},\mathbf{Y}$) coordinates can be reached by different elbow angle, depending on whether the subject elbow is resting on the table or not.

We utilized the Kalman filter for this task, which is an iterative process that uses a set of equations and discrete consecutive inputs to estimate the true value of the model being observed [25]. The Kalman filter assumes a linear relationship between the state **r**_*n*_ and the measurement **z**_*n*_ represented by

2 $${\mathbf{z}}_{n}={\mathbf{H}}_{n}{\mathbf{r}}_{n}+{\mathbf{Q}}_{n}$$

where ${\mathbf{H}}_{n}$ is a matrix that linearly relates the hand kinematics state **r**_*n*_ to the EMG signals **z**_*n*_ produced from the muscles and ${\mathbf{Q}}_{n}$ is the measurement noise.

The next state is computed using the system model:

3 $${{\varvec{r}}}_{n+1}={\mathbf{A}}_{n}{\mathbf{r}}_{n}+{\mathbf{w}}_{n}$$

where ${\mathbf{A}}_{n}$ is the coefficient matrix that linearly relates the current state to the next state and ${\mathbf{w}}_{n}$ is the process noise.

Although ${\mathbf{H}}_{n}$, ${\mathbf{A}}_{n}$, ${\mathbf{Q}}_{n}$ and ${\mathbf{w}}_{n}$ could be time-variant, we assume here that they are time-invariant to allow estimating them using the training data. The parameters **A** and **H** are estimated using the least-squares method as [18]

4 $$\mathbf{A}={\mathbf{R}}_{2}{\mathbf{R}}_{1}^{{\varvec{T}}}{({\mathbf{R}}_{1}{\mathbf{R}}_{1}^{{\varvec{T}}})}^{-1}$$

5 $$\mathbf{H}=\mathbf{z}{\mathbf{R}}^{{\varvec{T}}}{(\mathbf{R}{\mathbf{R}}^{{\varvec{T}}})}^{-1}$$

where

6 $$\mathbf{R}=\left(\begin{array}{ccc}{\mathrm{r}}_{\mathrm{1,1}}& \dots & {\mathrm{r}}_{1,\mathrm{M}}\\ \vdots & \ddots & \vdots \\ {\mathrm{r}}_{\mathrm{2,1}}& \dots & {\mathrm{r}}_{2,\mathrm{M}}\end{array}\right), \mathbf{R}1=\left(\begin{array}{ccc}{\mathrm{r}}_{\mathrm{1,1}}& \dots & {\mathrm{r}}_{1,\mathrm{M}-1}\\ \vdots & \ddots & \vdots \\ {\mathrm{r}}_{\mathrm{2,1}}& \dots & {\mathrm{r}}_{2,\mathrm{M}-1}\end{array}\right)$$

7 $$\mathbf{R}2=\left(\begin{array}{ccc}{\mathrm{r}}_{\mathrm{1,2}}& \dots & {\mathrm{r}}_{1,\mathrm{M}}\\ \vdots & \ddots & \vdots \\ {\mathrm{r}}_{\mathrm{2,2}}& \dots & {\mathrm{r}}_{2,\mathrm{M}}\end{array}\right), \mathbf{Z}=\left(\begin{array}{ccc}{\mathrm{z}}_{\mathrm{1,1}}& \dots & {\mathrm{z}}_{1,\mathrm{M}}\\ \vdots & \ddots & \vdots \\ {\mathrm{z}}_{\mathrm{4,1}}& \dots & {\mathrm{x}}_{4,\mathrm{M}}\end{array}\right)$$

The computed **A** and **H** are then used to estimate **W** and **Q** by

8 $$\mathbf{W}=\left({\mathbf{R}}_{2}-\mathbf{A}{\mathbf{R}}_{1}\right){\left({\mathbf{R}}_{2}-\mathbf{A}{\mathbf{R}}_{1}\right)}^{{\varvec{T}}}/(M-1)$$

and

9 $$\mathbf{Q}=(\mathbf{Z}-\mathbf{H}\mathbf{R}){(\mathbf{Z}-\mathbf{H}\mathbf{R})}^{{\varvec{T}}}/M$$

After the estimation of all Kalman filter parameters, Kalman filters are constructed for each subject and the obtained parameters are used to decode the elbow angle **θ**, and **X** and **Y** positions of the wrist using the measured EMG signal **z**_*n*_ using the following sequence.

- *Kalman filter time update equations*
  At each time instant *n,* we estimate the next state ${\widehat{\mathbf{r}}}_{n}^{-}$ using the prior state and the error covariance matrix ${\mathbf{P}}_{n}^{-}$ using the following equations:
  10 $${\widehat{\mathbf{r}}}_{n}^{-}=\mathbf{A}{\widehat{\mathbf{r}}}_{n-1},$$
  11 $${\mathbf{P}}_{n}^{-}=\mathbf{A}{\mathbf{P}}_{n-1}{\mathbf{A}}^{\mathbf{T}}+\mathbf{w}$$
  where initially, ${\widehat{\mathbf{r}}}_{n}^{-}$ and ${\mathbf{P}}_{n}^{-}$ are set to one.
- *Kalman filter measurement update equations*
  In the next step, we obtain the Kalman gain **k** and use it with the previous state ${\widehat{\mathbf{r}}}_{n}^{-}$ and the measured EMG **z**_*n*_ to correct the estimate computed earlier along with the error covariance matrix **H** using the following equations
  12 $${\mathbf{K}}_{n}={\mathbf{P}}_{n}^{-}{\mathbf{H}}^{\mathbf{T}}{(\mathbf{H}{\mathbf{P}}_{n}^{-}{\mathbf{H}}^{\mathbf{T}}+\mathbf{Q})}^{-1},$$
  13 $${\widehat{\mathbf{r}}}_{n}={\widehat{\mathbf{r}}}_{n}^{-}+{\mathbf{K}}_{n}\left({\mathbf{z}}_{n}-\mathbf{H}{\widehat{\mathbf{r}}}_{n}^{-}\right)\boldsymbol{ },$$
  14 $${\mathbf{P}}_{n}=\left(\mathbf{I}-{\mathbf{K}}_{n}\mathbf{H}\right){\mathbf{P}}_{n}^{-}.$$
  The previous equations represent a single iteration, where our kinematics parameters are being decoded for a time-window of 100 ms.

### Decoding methodology

**Fig. 13:**
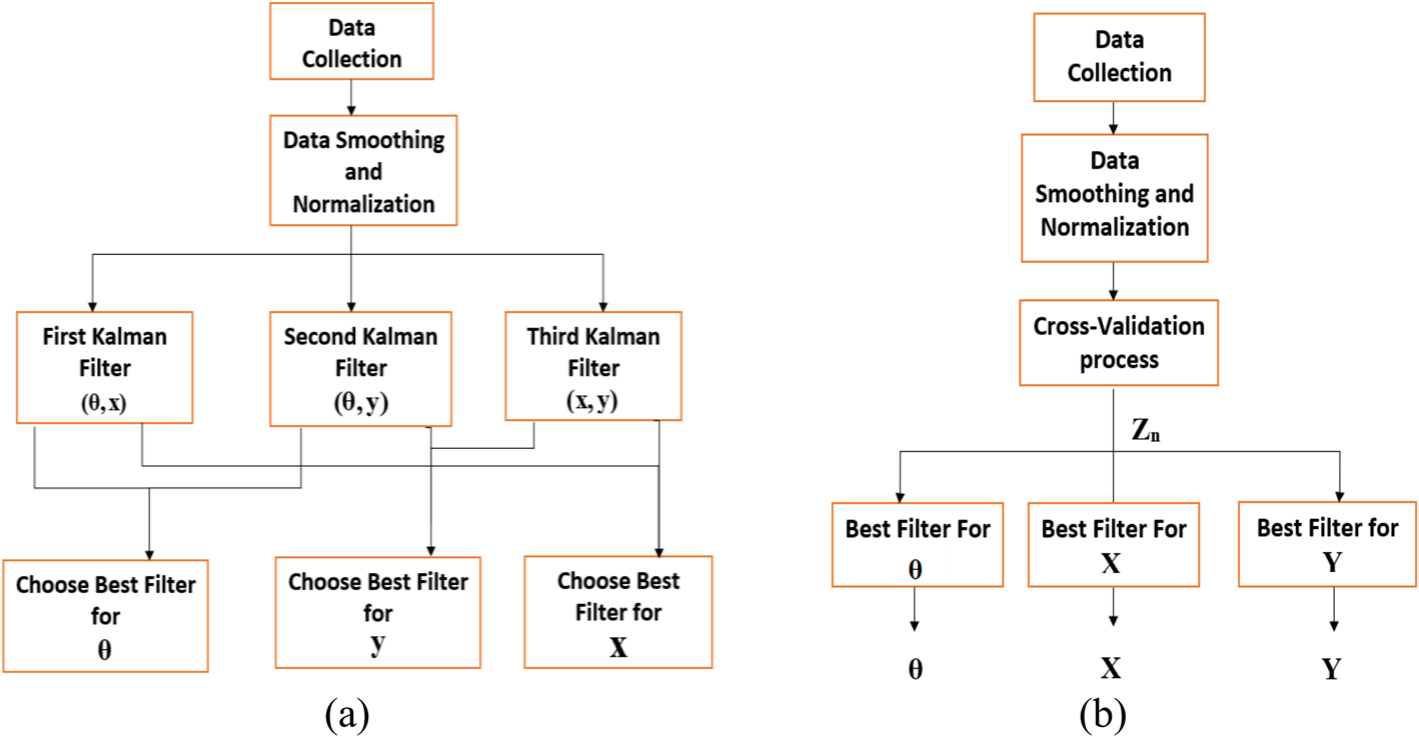
**a** Block diagram illustrating the cross-validation process for each subject to find the best decoding parameters combination. **b** Block diagram that shows the entire parameter combination decoding approach

In our study, we propose to decode our three parameters, **θ**, **X** and **Y** using a multi-Kalman filter approach. In this method, we construct two Kalman filters for decoding the three parameters. A cross-validation process is first performed on all data sets of each subject to decide which parameters combination is the most suitable. In this process, 20% of data were randomly selected to investigate which parameters combination results in better decoding performance. This data was then added to the training data, and used again as part of training data. Accordingly, for each subject, three Kalman filters are constructed in the validation step to test each pair of combinations. The two filters with the best combination results are then used in the decoding process. Figure 13 shows a graphical illustration of the cross-validation process, where we start with testing combinations (**θ**, **X)**, **(θ**, **Y)** and **(X, Y)**. We then investigate the decoding accuracy for each parameter and use the combination that results in the best performance for each parameter.

**Fig. 14:**
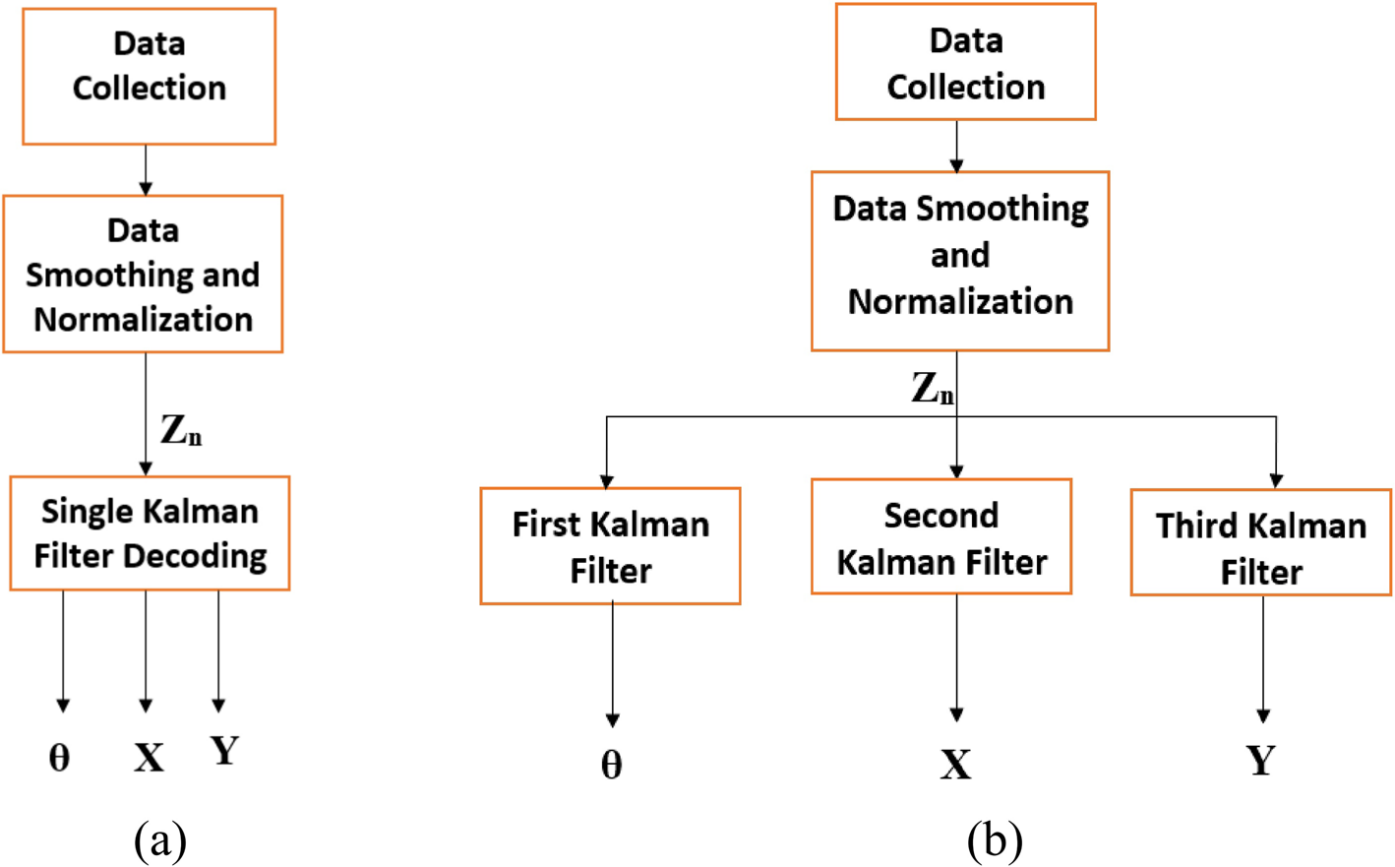
Block diagram showing **a** single and **b** three Kalman filters approaches

To compare, we also attempted to decode the three parameters using a single Kalman filter, where we use only one Kalman filter to decode all three parameters **θ**, **X**,** Y** using the measurement EMG **z**_*n*_. We also examined a three Kalman filter approach, where we construct a separate Kalman filter for each parameter to decode separately. Figure 14 shows a block diagram illustrating the steps of each of these two approaches.

### Performance evaluation

The correlation coefficient (CC) between the predicted movement and the actual movement is used as a performance measure metric given the linear relationship between the actual and predicted variables (see Additional file 1: Figs. S6 and S7), where the CC is estimated using Pearson’s formula [40]:

15 $$\mathrm{CC}=\frac{M\sum ab-(\sum a)(\sum b)}{\sqrt{\left[M\sum {a}^{2}-{(\sum a)}^{2}\right][M\sum {b}^{2}-{(\sum b)}^{2}]}}$$

where *M* is the number of samples, and *a* and *b* are the corresponding signals that we are testing their relation to each other (the actual and decoded kinematic movement parameters). In addition, root-mean-square error (RMSE) and normalized RMSE (NRMSE) were used:

16 $$\mathrm{RMSE}=\sqrt{\frac{{\sum }_{i=1}^{n}{(a-b)}^{2}}{N}}$$

17 $$\mathrm{NRMSE}=\frac{\mathrm{RMSE}}{\mathrm{max}\left(a\right)-\mathrm{min}(a)}$$

where *N* is the number of data samples.

## Supplementary Information

**Additional file 1.** Supplementary Performance Analysis and Comparison

## Data Availability

The data sets used and/or analysed during the current study are available from the corresponding author on reasonable request.
