# Supplementary material for: A multi-Kalman filter-based approach for decoding arm kinematics from EMG recordings
Source: Biomed Eng Online. 2022 Sep 3;21:60. doi: 10.1186/s12938-022-01030-6 (PMC9440508; doi:10.1186/s12938-022-01030-6)
Supplement: Supplementary file 1 — Additional file 1. Supplementary Performance Analysis and Comparison [file 12938_2022_1030_MOESM1_ESM.docx]

**Supplementary Information**

**Model Parameters:**

The training dataset was sued to estimate the 4 parameters A, H, W and Q of the Kalman filter. For each subject, we estimated the parameters for each KF combination (θ, X), (θ, Y) and (X, Y). Through the cross-validation phase, the best parameters were identified and subsequently used in the testing procedure. Fig. S1 shows the average value for all parameters across all subjects for (θ, X), (θ, Y) and (X, Y).

###

(a) (b) (c)

**Fig. S1** A heatmap showing the averaged parameters across all subjects for (a) (**θ**, **X**), (b) (**θ**, **Y**), and (c) (**X**, **Y**). The parameters represent the relationship between the kinematics **θ**, **X** and **Y** and the EMG of biceps (Bi), triceps (Tri), anterior deltoid (ADelt) and Lateral deltoid (LDelt). It also shows the system and measurement noise.

**Using Median Frequency (MDF) only for training and testing of Kalman filter:**

We computed the MDF from the EMG signal and used it to train and decode a Kalman filter instead of using the RMS amplitude presented in the main text. Fig. S2 shows a sample of the original and decoded kinematics for Subject 3. The figure demonstrates that the prediction is not able to track the actual kinematic and is not even within the values ranges of original input. Fig. S3 shows the CC and NRMSE for all subjects indicating a diminished performance for kinematics decoding achieving a CC of 0.0312 ± 0.107, 0.0010 ± 0.092 and 0.0174 ± 0.0977 and NRMSE of 0.811 ± 0.402, 0.743 ± 0.399 and 0.996 ± 0.599 for **θ**, **X** and **Y**, respectively.

(a) (b) (c)

**Fig. S2** A sample of the multi-Kalman filter performance using MDF only showing decoding results for (a) **θ**, (b) **X** and (c) **Y**.

(a) (b) (c)

**Fig. S3** CC and NRMSE for each subject using MDF for each of (a) **θ**, (b) **X** and (c) **Y.**

**Using combined MDF and RMS for training and testing of Kalman filter:**

We also examined using the MDF of the EMG signal in combination with the RMS proposed in the main text to train and decode the Kalman filter. Fig. S4 shows a sample of the original and decoded kinematics for Subject 3. The results demonstrate the success of the Kalman filter to follow the original kinematics. However, the performance was slightly below what is achieved using the RMS only. Fig. S5 shows the CC and NRMSE for all subjects. achieving a CC of 0.631 ± 0.117, 0.60 ± 0.20 and 0.574 ± 0.19, and a NRMSE of 0.837 ± 1.55, 1.12 ± 2.531 and 0.468 ± 0.52 for **θ**, **X** and **Y**, respectively.

(a) (b) (c)

**Fig. S4** A sample of the multi-Kalman filter performance using MDF and RMS combined showing the decoding result for (a) **θ**, (b) **X** and (c) **Y**.

(a) (b) (c)

**Fig. S5** CC and NRMSE for each subject using MDF and RMS combined for each of (a) **θ**, (b) **X** and (c) **Y** the subject-dependent test.

**Relationship between the original and predicted variables:**

In this section, we discuss the linearity of the relationship between the actual and the predicted kinematics. Fig. S6 shows the relationship between the actual and decoded **θ**, **X** and **Y** for Subject 1. The model used was linear regression achieving $r^{2}$ value of 0.468 ± 0.183, 0.4314 ± 0.166 and 0.478 ± 0.129 averaged across all subjects for **θ**, **X** and **Y**, respectively. Fig. S7 shows the detailed $r^{2}$ for each individual subject. The results demonstrate that the relationship between the actual and decoded kinematics is linear with the existence of minimal outliers.

1. (b)

(c)

**Fig. S6** A sample of actual vs decoded kinematics with fitted linear regression model for (a) **θ**, (b) **X** and (c) **Y**.

**Fig. S7** A detailed $r^{2}$ values computed between the actual and predicted **θ, X** and **Y** for each subject.
